# Successful selective arterial embolization in a patient with iliacus muscle hematoma and femoral neuropathy: A case report

**DOI:** 10.1097/MD.0000000000042097

**Published:** 2025-04-25

**Authors:** Sun Oh Kim, Jong-Seon Park

**Affiliations:** a Division of Cardiology, Yeungnam University Medical Center, Daegu, Republic of Korea.

**Keywords:** embolization, femoral neuropathy, iliacus muscle hematoma

## Abstract

**Rationale:**

Hematoma of the iliacus muscle is a rare but acknowledged complication associated with anticoagulant therapy. This condition may lead to nerve dysfunction due to compression, particularly affecting the femoral nerve. While there is no established guideline for its management, there has been a consensus favoring surgical intervention in cases of large hematoma with worsening neurological deficits.

**Patient concerns:**

A 67-year-old female presented to emergency room with acute groin pain, numbness in her right leg, and motor weakness following strenuous squat exercises. The patient had been prescribed warfarin for valvular atrial fibrillation. Laboratory tests revealed anemia and prolonged prothrombin time. Abdominal computed tomography revealed contrast leakage within the iliacus muscle. Subsequent electromyography and nerve conduction studies indicated findings suggestive of femoral neuropathy.

**Diagnosis:**

The patient was diagnosed with an actively bleeding iliacus muscle hematoma with femoral neuropathy.

**Intervention:**

Angiography of the right internal iliac artery identified contrast extravasation at the branch of the iliolumbar artery. Selective embolization of the iliolumbar artery branch was performed.

**Outcomes:**

Following embolization, no further decline in hemoglobin level was observed. With discontinuation of anticoagulants and conservative management, the patient’s sensory function in the right leg recovered, and the motor grade for knee extension improved from 1 to 4 within 3 weeks.

**Lessons:**

Even in the case of large iliacus hematoma with femoral neuropathy, conservative management without surgery can be a viable option if bleeding is well controlled with intervention.

## 1. Introduction

The iliopsoas muscle hematoma, although rare, is a recognized complication of anticoagulant therapy.^[1,2]^ In previous literature, the reported incidence of retroperitoneal hemorrhage varies from 1.3% to 6.6% in patients undergoing anticoagulation therapy.^[3]^ It is not only the most common site for spontaneous retroperitoneal hemorrhage but can also be affected by surgery, trauma, or bleeding from adjacent structures.^[4]^ Hematoma within or around the iliopsoas muscle can lead to complications such as hypovolemic shock, infection, severe pain, or nerve dysfunction.^[5]^ Regarding nerve dysfunction, the femoral nerve is particularly susceptible to compression due to its structural characteristics. The rigid fascia located distally to the iliacus and psoas muscles in the pelvic cavity can exacerbate the compressive effect of the hematoma.^[6]^ Goodfellow et al have explained that the psoas sheath and iliacus fascia are situated in separate compartments, with the tighter iliacus fascia more likely to create a high-pressure fluid collection around the femoral nerve.^[7]^ Femoral neuropathy, when involved, may manifest with various neurological symptoms. Sensory symptoms can range from mild paresthesia to complete numbness in the femoral nerve distribution, including the area innervated by its terminal branch, the saphenous nerve.^[8]^ Ongoing nerve compression can lead to motor dysfunction, with weakness or paralysis of the quadriceps and loss of patellar reflex.^[9]^ In this report, we present a case of an iliacus muscle hematoma with active bleeding, resulting in sudden femoral neuropathy.

## 2. Case report

**Figure 1.**
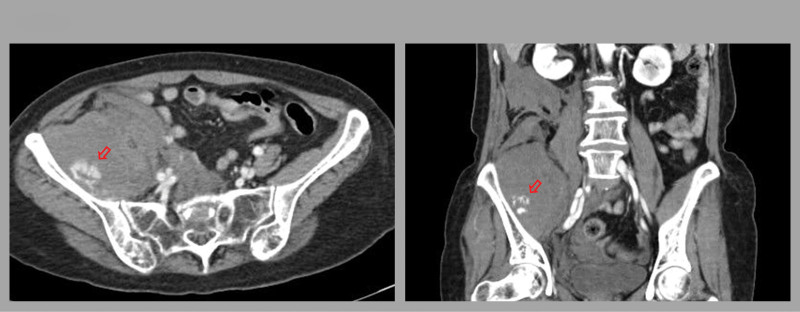
Contrast-enhanced abdominal computed tomography image of iliacus muscle hematoma. Fluid collection with contrast leakage (red arrow) was observed inside the right iliacus muscle (left: transverse view, right: coronal view).

A 67-year-old female presented to the emergency room with sudden groin pain, numbness in her right leg, and motor weakness persisting for 2 days. She experienced stabbing pain while performing 10 kg weight squat training, which progressed to worsening right leg motor weakness and increased pain over 3 hours. Her past medical history included atrial fibrillation, heart failure with reduced ejection fraction, a previous cerebrovascular accident, and dyslipidemia. Additionally, she had undergone percutaneous mitral balloon valvuloplasty for severe mitral stenosis, but still had progressive mitral stenosis and was taking 2 mg of warfarin daily. On arrival, her vital sign showed low blood pressure (90/60 mm Hg), a heart rate of 62 beats/min, respiratory rate of 16 breaths/min, and body temperature of 36.6ºC. Physical examination revealed right lower quadrant abdominal tenderness without rebound tenderness, with manual motor grades of 2 for hip flexion, 1 for knee extension, 4 for knee flexion, and 5 for ankle dorsiflexion. Laboratory tests showed leukocytosis (white blood cell count: 18,370/µL), mild anemia (hemoglobin [Hb] level 10.6g/dL), and an elevated international normalized ratio of 3.21. After 2 hours, her Hb level dropped to 8.8g/dL. Subsequent contrast-enhanced abdominal computed tomography (CT) revealed a fluid collection with contrast leakage inside the right iliacus muscle, indicating a hematoma with active bleeding, although the bleeding focus was not clearly identified on the CT image (Fig. 1).

**Figure 2.**
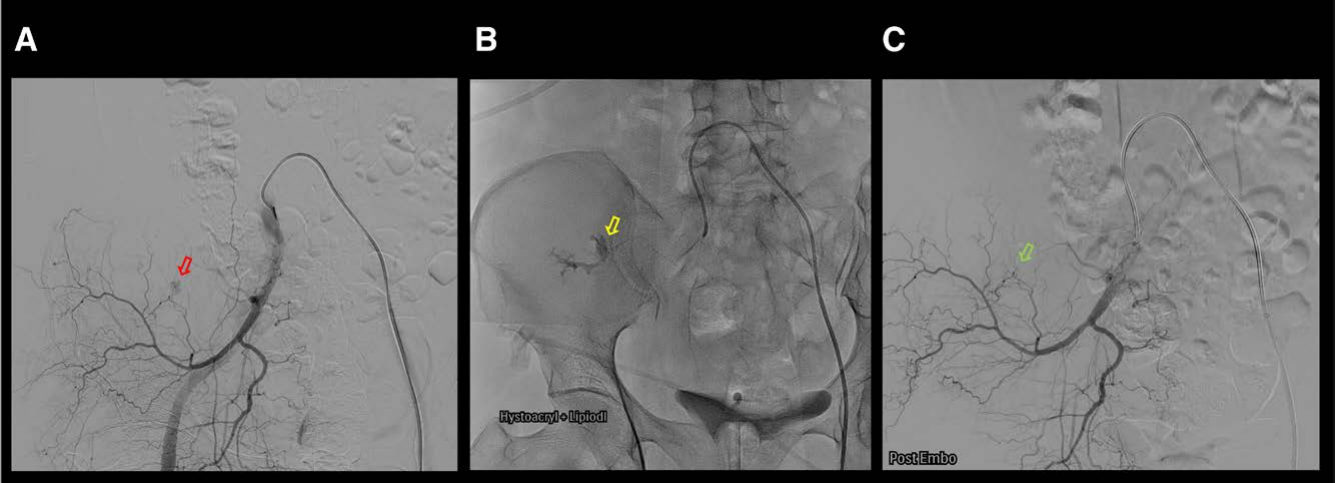
Selective embolization of the branch of the right iliolumbar artery. (A) Right internal iliac artery angiogram showed the contrast extravasation (right arrow) at the branch of the right iliolumbar artery. (B) Selective embolization (yellow arrow) was performed using a 0.3 mL 1:3 mixture of Histoacryl™ (B. Braun, Germany) and Lipiodol™ (Guerbet, French) via a microcatheter. (C) Post-embolization angiogram showed the disappearance of the contrast extravasation (green arrow).

**Figure 3.**
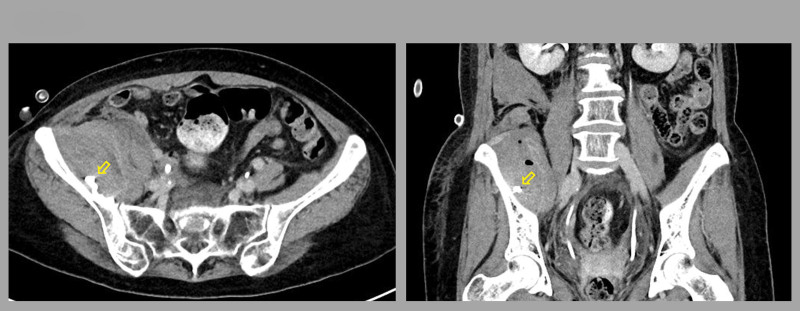
Contrast-enhanced abdominal computed tomography image of post-embolization state (yellow arrow, left: transverse view, right: coronal view).

Following the administration of vitamin K for warfarin reversal, peripheral artery angiogram was performed to identify the bleeding focus and facilitate embolization. The left common femoral artery was punctured, and a 0.035-inch “J” type guidewire (Glidewire™, Terumo, Japan) and 4-French Cobra catheter (Radifoucs™, Terumo, Japan) were used for angiography of the right internal iliac artery. Contrast extravasation was observed at a branch of the right iliolumbar artery (Fig. 2A). Arterial selection was performed with a 0.014-inch micro-guidewire (Streaming™, Asahi, Japan). Selective embolization was achieved using 0.3 mL of 1:3 mixture of Histoacryl™ (B. Braun, Germany) and Lipiodol™ (Guerbet, French) via a microcatheter (Caravel™, Asahi, Japan; Fig. 2B). Follow-up angiography confirmed the disappearance of the contrast extravasation (Fig. 2C). Four days later, follow-up abdominal contrast CT revealed a decreased amount of hematoma without contrast leakage (Fig. 3).

Due to persistent motor weakness, surgical treatment for nerve decompression was considered after embolization. However, the vascular surgeon cautioned about the risk of rebleeding after hematoma evacuation, the potential for embolic infarction due to cessation of warfarin for surgery, and given that the symptoms persisted beyond 72 hours, full recovery of nerve function was deemed unlikely. Finally, the patient declined surgery, leading to the decision to pursue conservative treatment. Absolute bed rest and maintenance of a low international normalized ratio (up to 1.5) were recommended.

Seven days after symptom onset, nerve conduction studies (NCS) and electromyography were conducted. Sensory NCS showed slightly reduced amplitude and velocity of sensory nerve action potentials of the right saphenous nerve, exceeding 50% compared to the left side. Motor NCS revealed low amplitude of compound muscle action potentials of the right femoral nerve (recorded at the vastus medialis, 13.11% compared to the left side). F-wave and H-reflex studies showed normal results. Needle electromyography demonstrated a reduced recruitment pattern in the right vastus medialis and iliopsoas, with abnormal spontaneous activity (positive sharp wave 1+, fibrillation potential 1+) observed in the right vastus medialis. These findings were indicative of femoral neuropathy.

As motor weakness gradually improved, no further interventions or surgical treatments were attempted. The patient commenced rehabilitation therapy and was discharged after 3 weeks with final manual motor grades of 4 + for hip flexion, 4 for knee extension, and 4 + for knee flexion. At a 1-month follow-up appointment, she was able to walk without difficulty.

## 3. Discussion

We present a case of iliacus muscle hematoma with active bleeding, resulting in femoral neuropathy, which was successfully managed by selective arterial embolization and conservative treatment.

Currently, there is no level 1 evidence guideline for the optimal treatment of iliacus muscle hematoma with femoral neuropathy due to the rarity of the disease.^[4,10]^ Treatment options include conservative management, intervention, and surgical decompression. Conservative treatment may involve correction of coagulopathy, transfusion, bed rest, analgesics, and bracing. Intervention options include percutaneous hematoma drainage for decompression or arterial embolization in cases of active bleeding.^[4,7,9]^

At present, consensus exists that small hematomas with minor neurologic deficits can be managed conservatively.^[4]^ However, large hematomas accompanied by severe pain or rapidly deteriorating neurologic function may necessitate surgical intervention. Some reports advocate for early surgical decompression, typically within 48 to 72 hours, in cases of iliopsoas muscle hematoma with femoral neuropathy to prevent the neurapraxia from progressing to axonal loss.^[10–13]^ Guild et al reported that delayed treatment, defined as intervention more than 48 hours after symptom onset, is associated with residual motor dysfunction in a significant proportion of patients. Additionally, conservative treatment alone resulted in poor nerve function outcomes.^[4]^ However, in real practice, performing early or even late surgical decompression can be challenging, especially for patients prescribed anticoagulants. Kim et al reported that among 25 cases of iliacus muscle hematoma, 9 patients were receiving anticoagulant or antiplatelet treatment, and only 4 patients were treated with surgical decompression, among them, only 1 patient fully recovered.^[14]^ With advancements in imaging modalities such as CT and ultrasonography and the difficulty of performing surgical decompression, recent trends favor intervention.^[4]^ Several reports advocate for using percutaneous drainage as first-line treatment.^[15–17]^ Additionally, if hematoma formation due to active bleeding is evident, arterial embolization can be considered first, rather than surgical decompression.^[17,18]^

In our case, the patient presented to the hospital more than 48 hours after the onset of symptoms. Within 72 hours of symptom onset, prompt transcatheter arterial embolization was performed. Following embolization, there was no worsening of the hematoma or nerve function. The discussion regarding surgical decompression was initiated well beyond 72 hours from the onset of symptoms. These factors led to the decision for conservative treatment, rather than additional intervention for decompression. This decision was driven by concerns about the surgical risk posed by the patient’s comorbidities, the risk of rebleeding, and uncertainty regarding nerve function recovery. Fortunately, the patient fully recovered after 4 weeks, likely due to the early intervention of active bleeding. Our case demonstrates that less invasive interventions and conservative treatments can be viable options for patients who have difficulty undergoing surgery due to multiple comorbidities.

## 4. Conclusion

Despite the rarity of the disease, retroperitoneal hematoma must be included in the complication of anticoagulation therapy for early recognition. Less invasive management should be considered first instead of surgical decompression. Further large-scale studies are needed to determine the extent to which intervention and conservative management can control the condition.

## Acknowledgments

This case report was written according to the CARE guidelines.

## Author contributions

**Writing – original draft:** Sun Oh Kim.

**Conceptualization:** Jong-Seon Park.

**Supervision:** Jong-Seon Park.

**Validation:** Jong-Seon Park.

**Writing – review & editing:** Sun Oh Kim, Jong-Seon Park.
